# Development of Acquired Immunity following Repeated Respiratory Syncytial Virus Infections in Cotton Rats

**DOI:** 10.1371/journal.pone.0155777

**Published:** 2016-05-25

**Authors:** Yoshiaki Yamaji, Yosuke Yasui, Tetsuo Nakayama

**Affiliations:** 1 Laboratory of Viral Infection I, Kitasato Institute for Life Sciences, Kitasato University, Shirokane 5-9-1, Minato-ku, Tokyo 108–8641, Japan; 2 Health Center, Keio University, Kanagawa 223–8521, Japan; University of Georgia, UNITED STATES

## Abstract

Respiratory syncytial virus (RSV) infections occur every year worldwide. Most infants are infected with RSV by one year of age and are reinfected because immune responses after the first infection are too weak to protect against subsequent infections. In the present study, immune responses against RSV were investigated in order to obtain a better understanding of repetitive RSV infections in cotton rats. No detectable neutralizing antibody (NT) was developed after the first infection, and the second infection was not prevented. The results of histological examinations revealed severe inflammation, viral antigens were detected around bronchial epithelial cells, and infectious viruses were recovered from lung homogenates. Following the second infection neutralizing antibodies were significantly elevated, and CD8^+^ cells were activated in response to RSV-F_253-265_. No viral antigens was detected thereafter in lung tissues and infectious viruses were not recovered. Similar results were obtained in the present study using the subgroups A and B. These results support the induction of humoral and cellular immune responses following repetitive infections with RSV; however, these responses were insufficient to eliminate viruses in the first and second infections.

## Introduction

Respiratory syncytial virus (RSV) infections are a common cause of lower respiratory diseases in infants and the elderly worldwide. Newborn infants, high-risk individuals, and the elderly are susceptible to RSV infections [1, 2]. RSV infections mainly cause upper respiratory illnesses, with the subsequent development of lower respiratory symptoms, such as wheezing, dyspnea, and, ultimately, respiratory failure in some very young infants. Approximately 33.8 million infants are estimated to have been infected with RSV, with 66,000–199,000 deaths annually [3]. Furthermore, a 3.2-fold increase in the risk of wheezing and 3.1-fold increase in the risk of asthma have been reported in hospitalized patients with RSV bronchiolitis [4]. RSV bronchiolitis is associated with the infiltration of neutrophils and eosinophils in lung tissues, and may be related to Th2-type cytokine responses. Legg et al. [5] analyzed the nasal lavage fluid from patients, and demonstrated that IFN-γ levels were significantly lower in infants who developed acute bronchiolitis after RSV infection on Day 1–2 than in infants who did not developed acute bronchiolitis. They also analyzed cytokine mRNA production by stimulated peripheral blood mononuclear cells (PBMC), and demonstrated IL-4/ IFN-γ mRNA production was significantly higher in the infants who developed acute bronchiolitis.

Most infants are infected with RSV until three years of age and are reinfected after recovery. The reason why infants are repeatedly infected has been attributed to the poor immune responses induced by the initial RSV infection [6–8]. Neutralizing (NT) antibodies against RSV have been suggested to bind to the RSV-fusion (F) and attachment glycoprotein (G) proteins, which play important roles in protecting against infections. The RSV-F protein is genetically stable among wild circulating strains and is crucially involved in the infection process of virus-cell fusion. A humanized monoclonal antibody preparation against the F protein, called palivizumab (Synagis®: MedImmune, Gaithersburg, MD), is currently used for prophylaxis against RSV infection.

Immunological studies after natural RSV infection have been conducted using experimental animal models since the 1980s. Prince et al. [9] reported that virus clearance was promoted by the administration of a high dose of anti-RSV serum to infant cotton rats. However, difficulties are associated with the induction of NT antibodies against RSV following natural infection in young infants. Murphy et al. [10, 11] showed that infants infected with RSV produced moderate levels of IgG and IgA following primary infection, while one infant failed to develop EIA antibodies against the RSV-F protein. Although the reason for this phenomenon currently remains unknown, plausible mechanisms have included the presence of maternally conferred antibodies that interfere with immune responses to RSV infection in infants due to binding to RSV particles [10]. NT antibodies protect against viral infections, and the cellular immunity of cytotoxic lymphocytes (CTL) eliminated RSV-infected cells. However, CTL activity has not yet been investigated in detail in a clinical setting because of the difficulties associated with sample collection and assay procedures.

In order to obtain a better understanding of NT and CTL responses in young infants, the development of immune responses was examined in cotton rats infected several times with RSV subgroups A and B.

## Materials and Methods

### Study design

This study protocol was approved by the Committee on the Ethics of Animal Experiments of the University of Kitasato Institute for Life Sciences (Permit Number: 13–003). All procedure was performed under sodium pentobarbital anesthesia, and all efforts were made to minimize suffering.

### Cells and viruses

RSV/A/Tokyo/2012 and RSV/B/Tokyo/2012 were isolated from clinical samples in 2012. RSV/A/Tokyo/2012 was propagated in HEp-2 cells and RSV/B/Tokyo/2012 was propagated in Vero cells. HEp-2 cells were maintained in minimum essential medium (MEM) supplemented with 10% fetal bovine serum (FBS). Vero cells were maintained in Eagle’s MEM supplemented with 5% FBS.

### RSV infection in cotton rats

**Fig 1:**
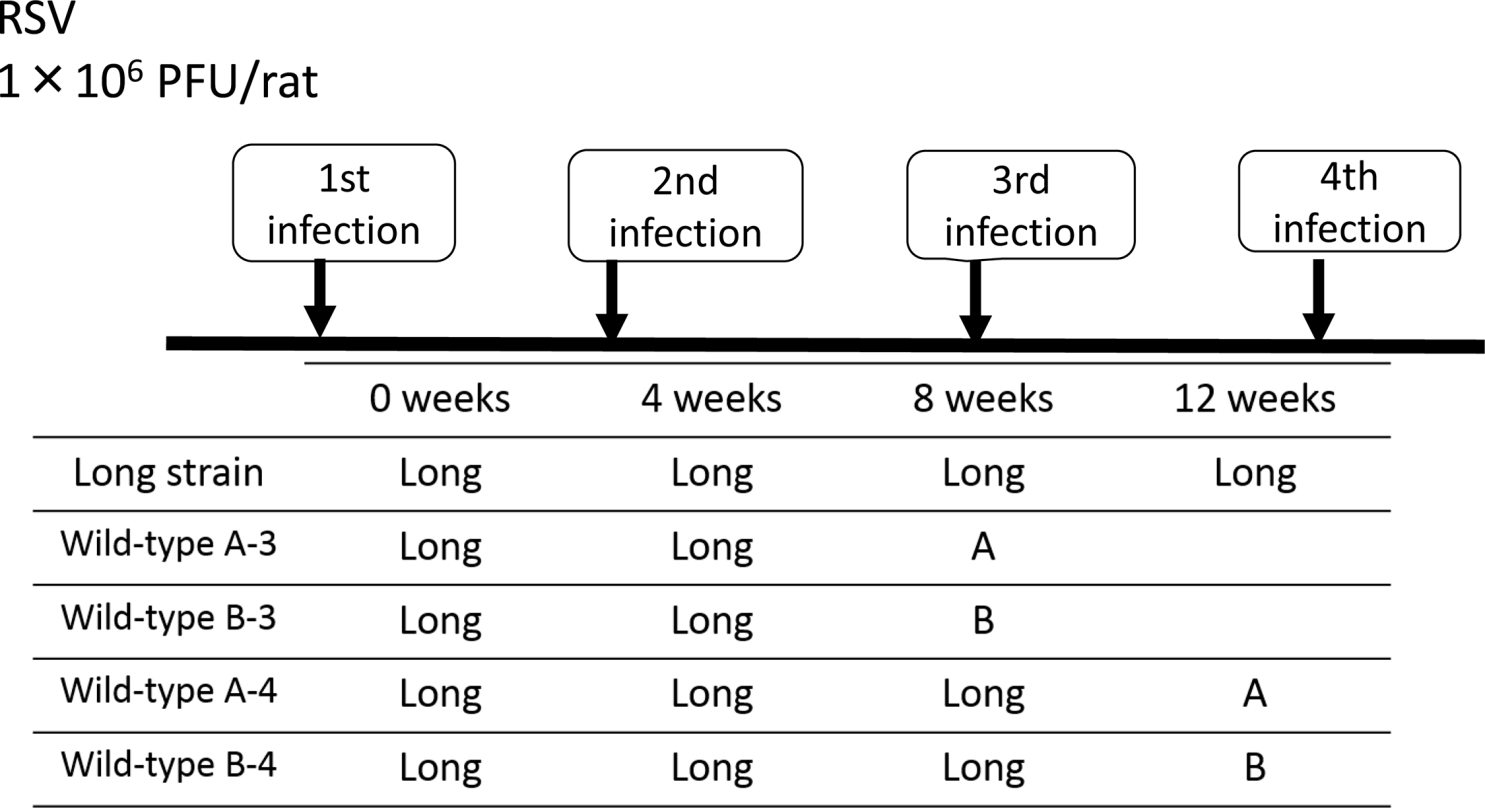
Experimental schedule for repetitive respiratory syncytial virus infections. Cotton rats were infected with the Long strain (1×10^6^ pfu/rat). Three cotton rats each were sacrificed 4 days after the first, second, third, and fourth infections. The third and fourth infection were analyzed twice. In order to examine immune responses to subgroup A and B, twelve cotton rats were prepared and divided into four groups. Six rats were challenged with RSV/A/Tokyo/2012 or RSV/B/Tokyo/2012 one month after the second infection, and sacrificed the groups of wild-type A-3 and B-3. The other rats were challenged one month after the third infection, and sacrificed. Wild-type A-3, B-3, A-4, and B-4 are showing a third and fourth infection with RSV/A/Tokyo/2012 or RSV/B/Tokyo/2012.

The study protocol is shown in Fig 1. Cotton rats (*Sigmodon hispidus*), aged 7–8 weeks old, were prepared for repeated RSV infections with the Long strain, and three animals were included in each group for the first, second, third, and fourth infection. The experiments of the third and fourth infections were performed twice. Cotton rats were anesthetized and then infected with the RSV Long strain (1×10^6^ pfu/rat) through an intranasal route at 0, 4, 8, and 12 weeks. Cotton rats were sacrificed 4 days after each infection. In order to examine immune responses to the subgroups A and B, twelve cotton rats were prepared and divided into four groups. Two groups, showing A-3 and B-3, were challenged with RSV/A/Tokyo/2012 or RSV/B/Tokyo/2012 one month after the second infection, and sacrificed. The other two groups, showing A-4 and B-4, were challenged one month after the third infection, and sacrificed. Serum samples were obtained immediately before infection [12].

### Flow cytometry

A total of 1–2×10^6^ freshly isolated splenocytes was stimulated with 1 μM of individual peptides in the presence of 10 μg/ml brefeldin A (Alomone labs, Israel) in 500 μl culture medium (RPMI, supplemented with 10% FCS) at 37°C for 5 hours. Peptides corresponding to CD8^+^ T cell epitopes were synthesized (Medical … Biological Laboratories Co. Ltd. Japan) [13, 14]. After the stimulation with 1 μM of respective peptides, cells were harvested, washed, and incubated with an anti-CD8 antibody (R&D Systems, USA) for 30 min. Cells were fixed with the Cytofix/cytoperm kit (BD Pharmingen, San Diego, CA), and intracellular cytokines were stained with a goat IgG antibody against cotton rat IFN-γ (R&D Systems, USA) and anti-goat IgG-PE-Cy7 (Santa Cruz Biotechnology, Inc., USA) for 60 min. Cellular populations were analyzed using flow cytometry with *Cytomics* FC 500 (Beckman Coulter, Inc., USA) [15]. The splenocytes were counted 100,000 cells or for six minutes, and CD8^+^ IFN-γ^+^ cells were analyzed in gate.

### NT antibody responses

NT against RSV was performed with the 50% plaque reduction assay using the Long strain, as previously reported [16]. Briefly, serum samples were serially diluted two-fold, starting from a 1:10 dilution, and then mixed with an equal volume of RSV subgroup A (100 PFU) at room temperature for one hour. In the present study, NT antibody titers were examined not only against the Long strain, but also against wild strains. Serum samples were serially diluted two-fold, starting from a 1:5 dilution, and then mixed with an equal volume of RSV subgroup B (100 TCID_50_) at room temperature for one hour. The mixture was incubated on Vero cells at 37°C for 6 days in 5% CO_2_. These cells were fixed with 99.5% ethanol for 10 minutes and washed with PBS. Polyclonal antibodies against RSV (Abcam, Cambridge, UK) were diluted at 1:1,000 and incubated at room temperature for two hours. The antibodies were removed and cells were washed with PBS. Anti-goat IgG-HRP (Santa Cruz Biotechnology, Inc.), diluted at 1:1,000, was then added, and the plate was incubated at room temperature for 40 minutes. The plate was washed with PBS, and Vero cells were stained using the Peroxidase Stain DAB Kit with a Metal Enhancer DAB stain (NACALAI TESQUE, INC. Kyoto, Japan). NT antibody titers were expressed as the reciprocal of the highest dilutions that inhibited the appearance of the CPE of RSV.

### HE staining and immunostaining

Lung tissues were fixed with 4% formaldehyde, embedded in paraffin, sectioned, and stained with hematoxylin-eosin (HE). Immunostaining was performed using a four-clone blend of monoclonal antibodies against the RSV P, F, and N proteins (AdB Serotec, UK) and anti-mouse IgG conjugated with HRP (Dako North America, Inc.)[17]. Lung tissue specimens were stained using the Peroxidase Stain DAB Kit and Metal Enhancer DAB stain (Nacalai Tesque, Inc., Kyoto, Japan).

As for the scoring of histological findings, six random fields per group were scored for histopathology using the following criteria: 1, swelling of alveolar walls; 1, destruction of bronchial epithelial cells; 1, peribronchial infiltration of inflammatory cells; 2, mucus as a production cause of occlusion in the bronchial space; and 1, bleeding. Microscopic images were taken with a Life Technologies EVOS XL Core light microscope at 40× magnification.

### Detection of infectious RSV in rat lungs

Cotton rats were sacrificed four days after being infected and lung tissues were obtained in order to detect infectious RSV. A total of 0.1 ml of a 10-fold serial dilution of the lung tissue homogenate was placed on HEp-2 cells, which were then incubated at 37°C with shaking every 30 min for 4 hours. MEM 5% FBS with 0.5% agar was overlaid. Plaque numbers were counted after an incubation at 37°C for 6 days, and infectivity was expressed as the number of plaques adjusted to 1 g of lung tissue, as described previously[17, 18].

## Results

### NT antibody responses following RSV infections

**Fig 2:**
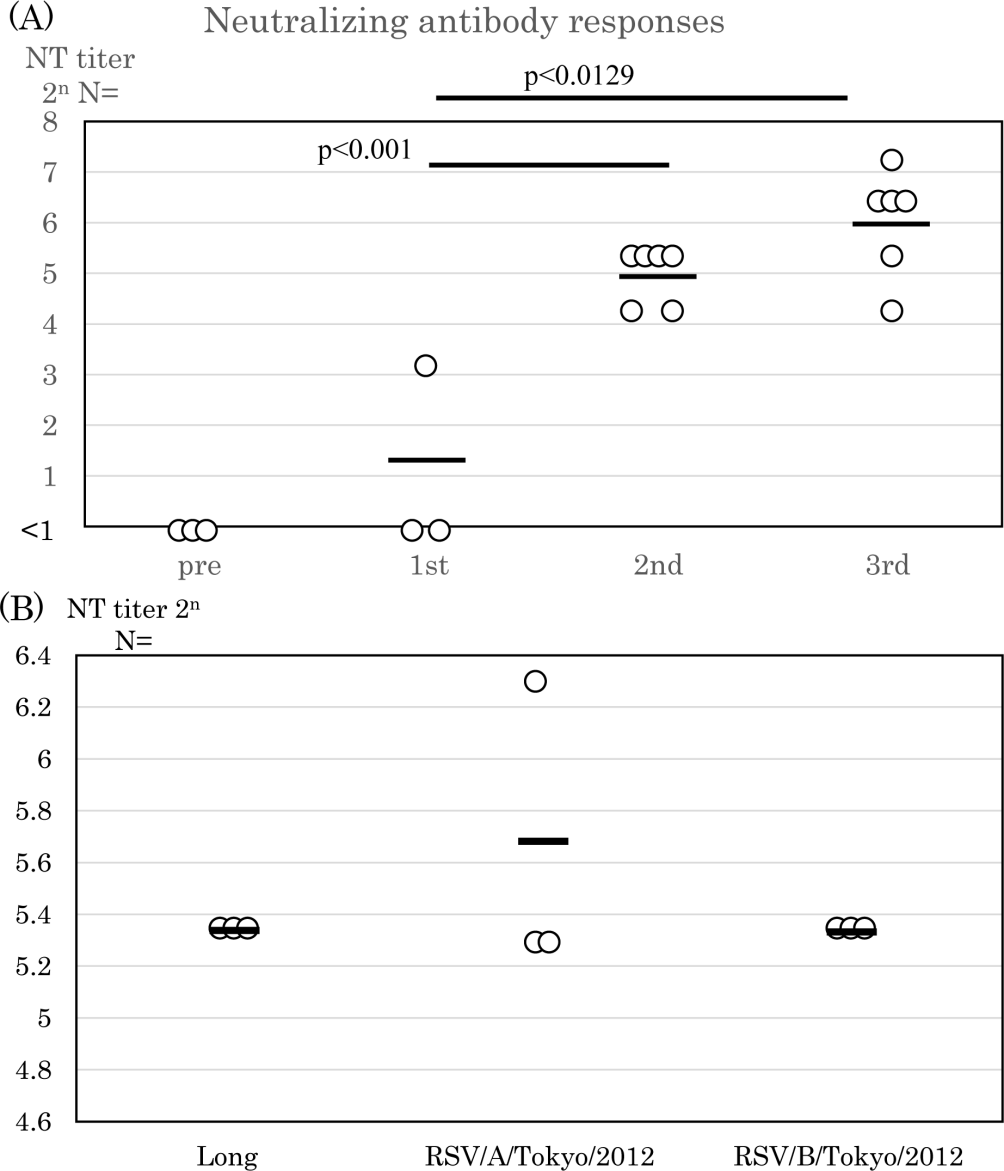
Neutralizing antibody responses against RSV. (A) Serum samples were obtained 4 weeks after the first, second, and third infections. Neutralizing (NT) antibody responses were examined using the 50% plaque reduction assay with the Long strain. Bars show the average (pre and first infection: n = 3, second and third infections: n = 6 rats per group). Three serum samples were obtained 4 weeks after the first infection, and six samples were collected after subsequent infections. (B) Serum samples obtained one month after the third infection were used. Serum samples were mixed with RSV/A/Tokyo/2012 or RSV/B/Tokyo/2012, incubated at room temperature, and inoculated on Vero cells. They were stained with polyclonal antibodies against RSV conjugated with HRP. Data are shown as the average of three animals per group.

The results of the development of NT antibodies are shown in Fig 2A and Fig 2B. NT antibody titers against the Long strain were below the detection limit one month after the first infection. NT antibody titers increased to 2^4.98±0.51^ one month after the scond infection, and to 2^5.98±1.03^ following the third infection (Fig 2A). NT antibody responses against different subgroups were examined. In the present study, the wild-type clinical isolates, RSV/A/Tokyo/2012 and RSV/B/Tokyo/2012, were used, and the development of NT antibodies against different subgroups is shown in Fig 2B. NT antibody titers against RSV/B/Tokyo/2012 increased to 2^5.32^ following the third infection, similar to those against the Long strain and wild strain. These results suggest that cross-reactive NT antibodies were induced by repetitive infections with RSV.

### Assessment of the ratio of CD8^+^ IFN-γ^+^ cells

**Fig 3:**
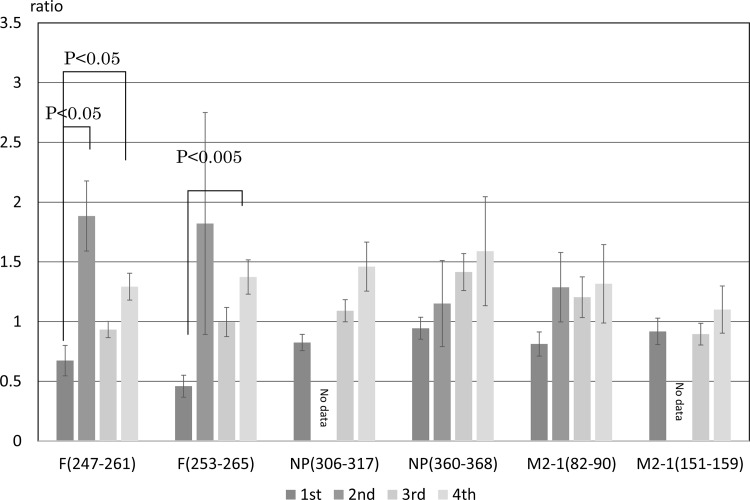
Ratio of CD8^+^ INF-γ^+^ cells in splenocytes in response to M2-1, F, and NP peptides. Cotton rats were infected with the RSV Long strain and splenocytes were obtained four days after the first, second, third, and fourth infections. Data are shown as the average of three (at the first and second infections) to six (at the third and fourth infections) animals. The number of cells was counted in gate. Data show the ratio of the number of cells stimulated with the antigen divided by the number of cells incubated in RPMI buffer. Bars represent means ± SE.

CTL responses were assessed by counting the number of CD8^+^ IFN-γ^+^ cells in spleen cell cultures stimulated with peptides using flow cytometry. The results obtained show the ratio of the number of CD8^+^ IFN-γ^+^ cells stimulated with the antigen divided by that obtained without the stimulation. The results for first infection groups showed a ratio of 1.0 or lower, indicating that CD8^+^ IFN-γ^+^ cells had not responded to peptides after the first infection. The number of CD8^+^ IFN-γ^+^ cells increased with repetitive infections (Fig 3). In the case of the stimulation with F (247–261), the number of CD8^+^ IFN-γ^+^ was increase 1.8-fold higher than that without the stimulation following the second infection. This result demonstrated that RSV-specific cells were induced after the second infection. The results obtained following the third infection, revealed that the number of CD8^+^ IFN-γ^+^ cells stimulated with F (247–261) or F (253–265) was lower than that following the second infection. The number of CD8^+^ IFN-γ^+^ cells in response to NP (360–368) increased by 1.46±0.50-fold, while that in response to NP (306–317) increased by 1.59±1.12-fold following the fourth infection. The number of CD8^+^ IFN-γ^+^ cells in response to M2-1 was lower than that in cells stimulated with F and NP. The peptides used in the present study were selected based on the previous reports [19, 20, 21]

### Viral clearance

**Fig 4:**
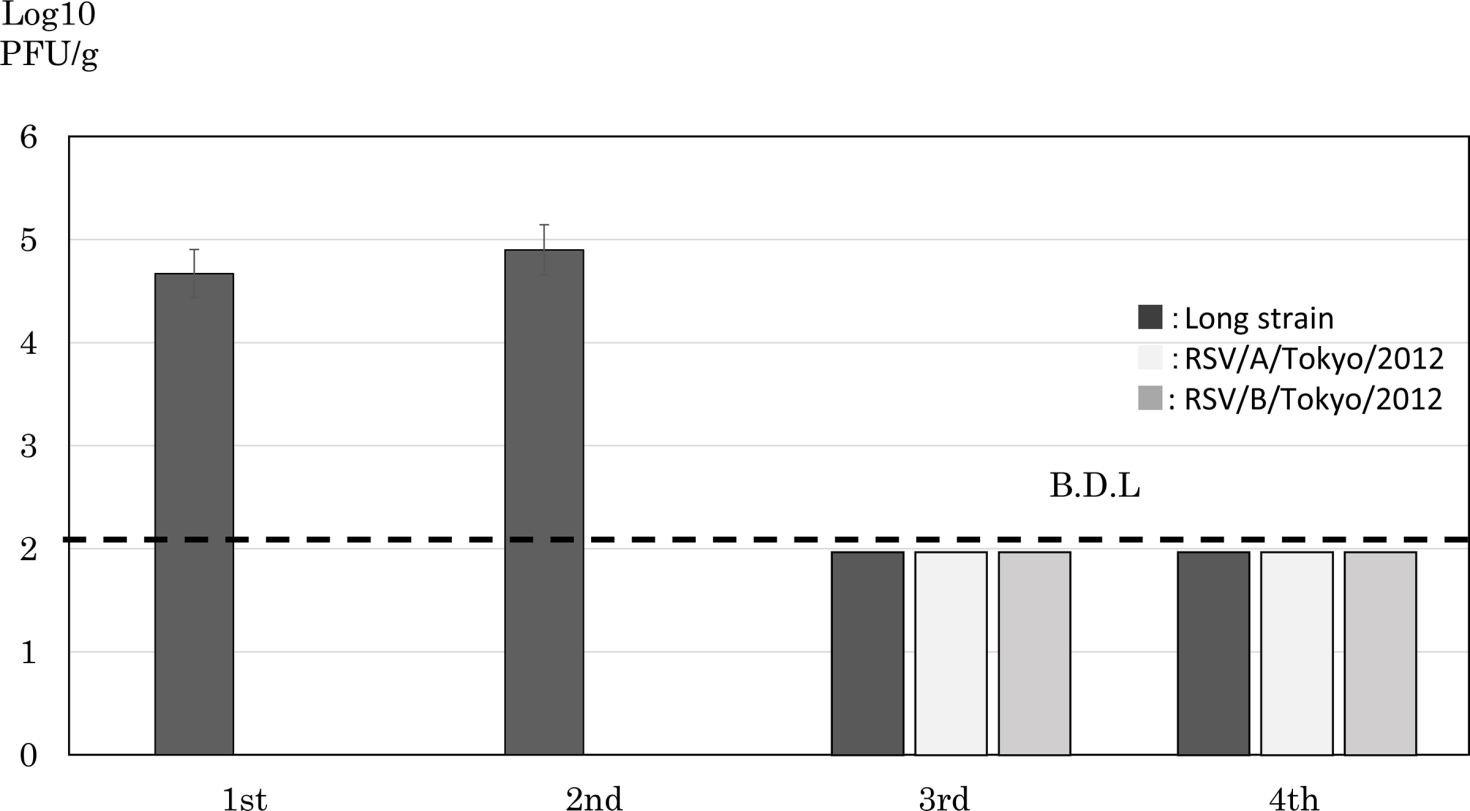
Recovery of the infectious RSV Long strain from lung tissues. Three cotton rats were infected with 1.0×10^6^ PFU of the RSV Long strain. Virus infectivity was monitored in lung homogenates, and RSV infectivity was shown as PFU per 1 gram of lung tissue. B.D.L indicates below the detection limit.

The infectious titer of the virus recovered from the lungs was expressed as PFU per gram of lung tissues. A total of 10^4.6^ PFU/g of infectious viruses was detected in the lung homogenates obtained after the first and second infections (Fig 4). No infectious virus was detected in lung tissues obtained after the third or fourth infection. Similar results were obtained in an examination of infections with RSV/A/Tokyo/2012 and RSV/B/Tokyo/2012. These results suggest that animals acquired protective immune responses with repetitive infections using the same strain, and support the induction of cross-reactive NT antibodies (Fig 2B).

### Histopathological findings

**Fig 5:**
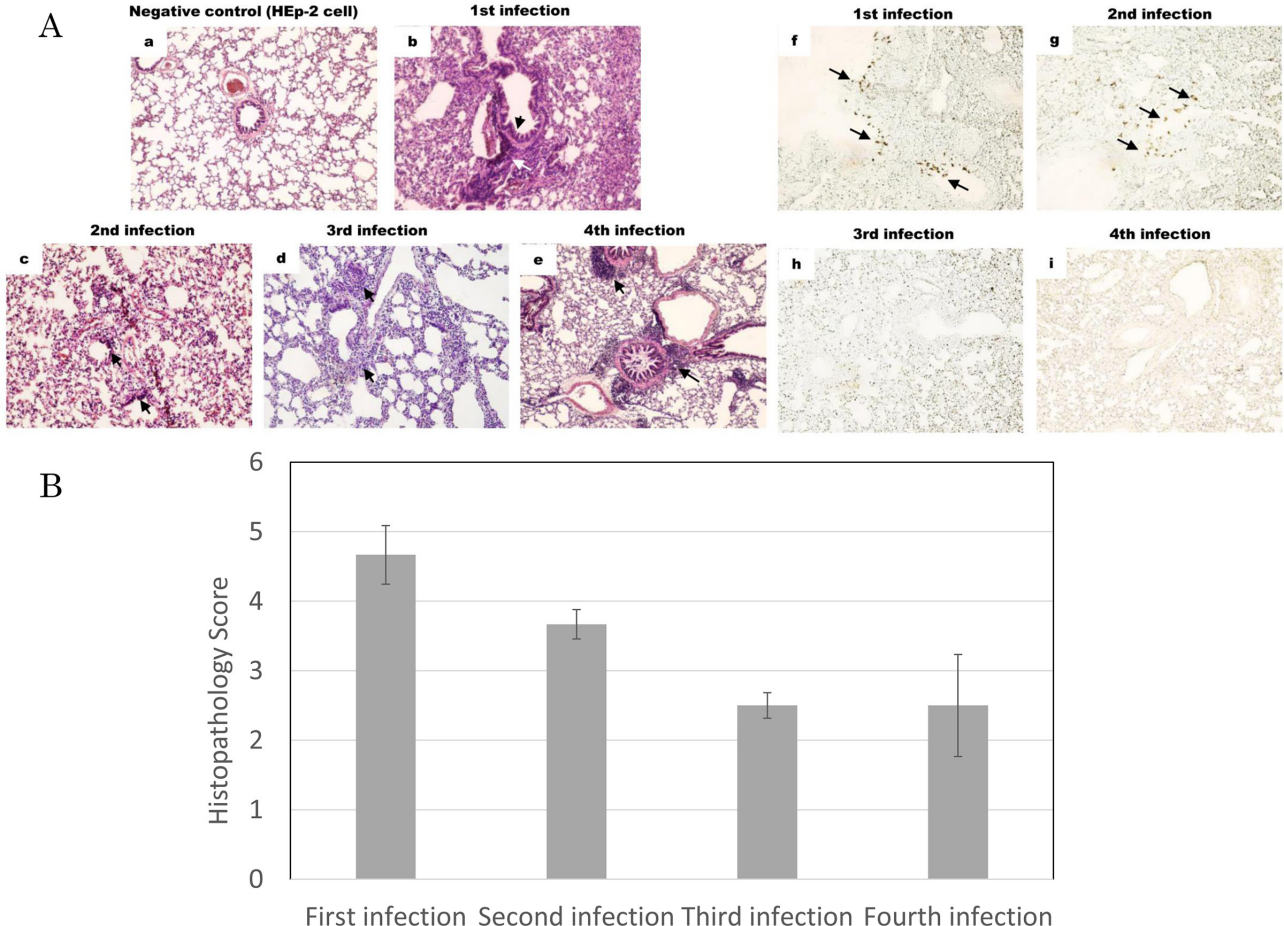
Histopathological findings of lung tissues following infections with the RSV Long strain. (A) The results of hematoxylin-eosin (HE) staining and immunostaining are shown. Lung tissues were obtained from cotton rats inoculated with the supernatant of the HEp-2 cell homogenate through an intranasal route as a negative control (a). Lung tissues were obtained four days after the first, second, third, and fourth infections and the results of HE staining are shown (b-e). Lung tissues were stained with a four-clone blend of monoclonal antibodies against the RSV P, F, and N proteins and anti-mouse IgG conjugated with HRP (f-i). Each panel shows the magnification of 1:200. (B) Lung tissues infected with the Long strain were scored. Lung tissues were sectioned and stained with HE. Six random fields per group were scored for histopathology based on the following criteria: narrowing alveolar space, thickness of alveolar walls, destruction of bronchial epithelial cells, peribronchial infiltration of inflammatory cells, mucus production, and bleeding. Bars represent means ± SE.

Inflammatory cells were observed in lung tissues after each infection (Fig 5A). The thickening of alveolar walls and infiltration of inflammatory cells were less severe following the fourth infection (Figs 5A b-e). RSV antigens were detected around the bronchioles after the first and second infections (Fig 5A f and g), but were absent after the third and fourth infections (Fig 5A h and i). The histological score was very high following the first infection (Fig 5B). The swelling of alveolar walls, severe destruction of bronchial epithelial cells, peribronchial infiltration of inflammatory cells and, bleeding were detected in the fields examined. The histological score decreased following the third and fourth infections.

**Fig 6:**
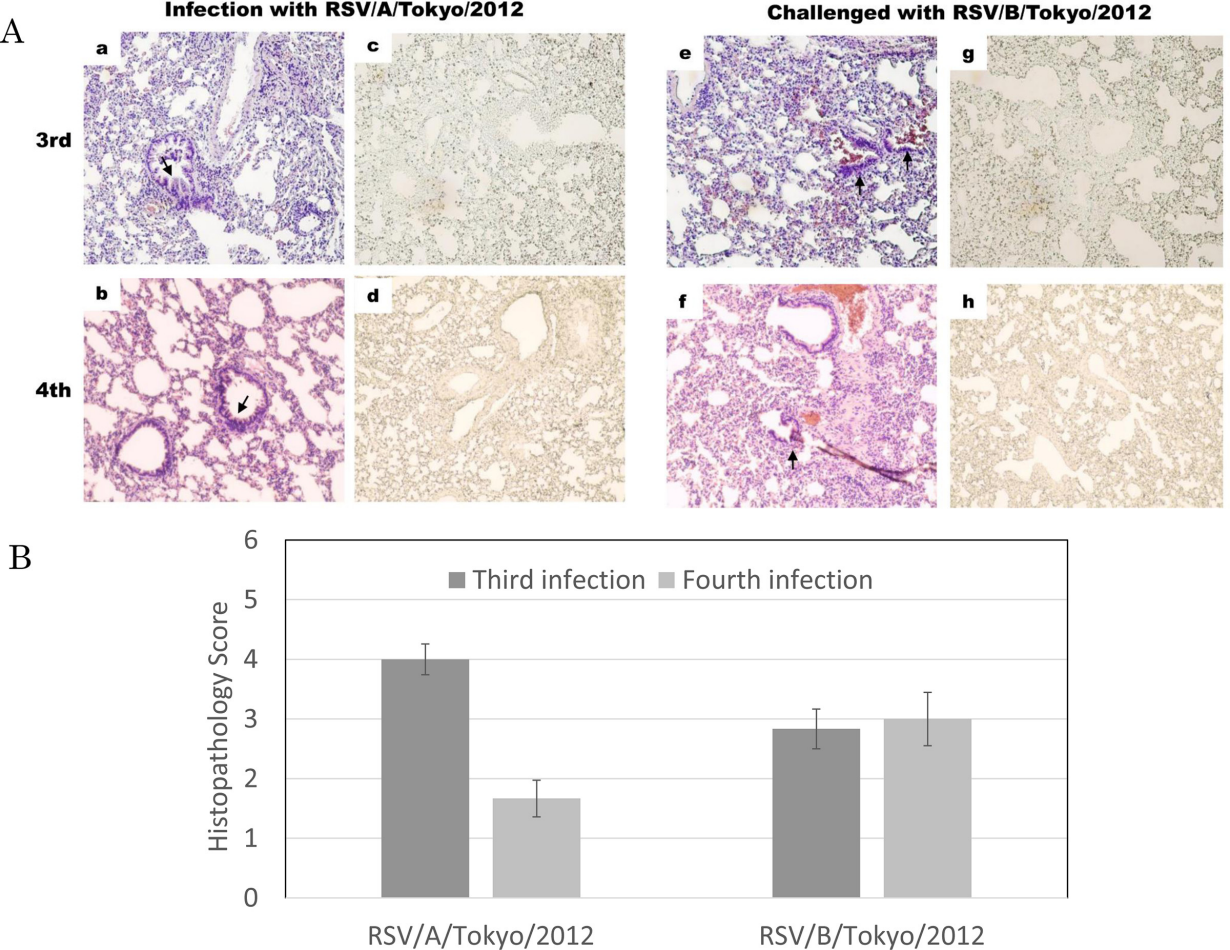
**Histopathological findings after third and fourth infections with wild-type RSV subgroups A and B.** (A) Cotton rats were infected with RSV/A/Tokyo/2012 or RSV/B/Tokyo/2012 in the second or third infections with the Long strain. The results of HE staining after infections with RSV/A/Tokyo/2012 (a and b), and those of immunostaining after infections with RSV/A/Tokyo/2012 were shown (c and d). The results of HE staining after infections with RSV/B/Tokyo/2012 were shown (e and f), and those of immunostaining after infections with RSV/B/Tokyo/2012 were shown (g and h). Each panel shows a magnification of 1:200. (B) Lung tissues infected with RSV/A/Tokyo/2012 or RSV/B/Tokyo/2012 were scored. Bars represent means ± SE.

Cross-reactive NT antibodies were induced after the third infection with the Long strain (Fig 2B). No infectious virus was recovered after the third or fourth infection with RSV/A/Tokyo/2012 and RSV/B/Tokyo/2012 (Fig 4). The histological findings of both groups infected with RSV/A/Tokyo/2012 and RSV/B/Tokyo/2012 are shown in Fig 6A. Animals were infected with RSV/A/Tokyo/2012 and developed pneumonia; however no RSV antigens were detected on the field following the third infection (Fig 6A a and c). In the case of the fourth infection, although very mild pneumonia was observed in lung tissues (Fig 6A b), no RSV antigens were detected around the bronchioles of all animals (Fig 6A d). The infiltration of inflammatory cells and histological findings of pneumonia were observed following the third and fourth infections with heterologous RSV/B/Tokyo/2012 (Fig 6A e and f). However, no RSV antigens were detected (Fig 6A g and h). These results were consistent with the clearance of infectious viruses from the lungs. Histopathological findings were scored in order to assess the severity of inflammation. Histopathological scores were lower in the third and fourth infections than in the first and second infections (Fig 5B). The same results were obtained following infections with RSV/A/Tokyo/2012 and RSV/B/Tokyo/2012 (Fig 6B). These results support repetitive infections contributing to milder inflammation.

## Discussion

RSV causes repetitive respiratory infections in young infants because of poor immune responses to the initial infection. Therefore, infants acquire strong and cross-reactive immune responses through repeated infections. Premature immune responses are regarded as one of the reasons for insufficient immune responses after the first infection, and maternally conferred antibodies interfere with their immune responses by binding virus particles. Dendritic cells and macrophages do not induce strong immune responses because RSV has been shown to disturb the immune system [7, 8].

In the present study, NT antibody titers increased significantly after the second RSV infection (Fig 2). The acquisition of a moderate or high NT antibody titer is needed for protection against RSV infections in animal models [9]. NT antibody titers were elevated after the second infection, with animals being protected against the third and fourth infections. However, other studies have indicated that animals produced IgG and sufficient NT antibody responses after RSV infection in mouse model [22, 23]. These differences have been attributed to differences in the animal species examined and experimental methods used. Repetitive infections with the Long strain induced cross-reactive immune responses against RSV/B/Tokyo/2012, with similar NT antibody titers being noted against RSV/A/Tokyo/2012 (Fig 2).

Th1 immune responses are important for protection against viral infections. A formalin-inactivated RSV (FI-RSV) vaccine was developed in the 1960s that paradoxically enhanced the infiltration of inflammatory cells into the lungs of vaccine recipients following subsequent natural infections with RSV. Th1-type immune responses inhibited the infiltration of inflammatory cells into the lungs following RSV infections [13, 24]. In a vaccine study, the induction of IFN-γ was used as a biological marker for the inhibition of eosinophilia and induction of Th1 immune responses. In the present study, RSV-specific CD8^+^ cells were induced after the second infection and responded to RSV-F and NP (Fig 3). However, mild pneumonia was observed in the lungs following the third and fourth infections. The immune responses induced by the first or second infection was insufficient to inhibit inflammation following subsequent infections. However, no infectious viruses were detected in the lungs following the third and fourth infections (Fig 4). Recombinant viruses expressing the RSV-F or G protein have been developed, induce RSV-specific antibodies, and inhibit RSV infections in cotton rats [17, 18, 25]. These findings indicate that RSV infection is prevented by the induction of antibodies against the RSV-F and G proteins. However, some issues have not yet been sufficiently elucidated, such as immunizations using RSV-F and G proteins not being effective against the different subgroups, and inflammatory cells not being completely cleared from the peribronchial region. In the present study, repetitive infections with RSV induced immune responses against both subgroups. Animals may eliminate RSV through the induction of antibodies and RSV-specific CD8^+^ cells. This result supports the clearance of both subgroups in cotton rats through the induction of CTL activity. A previous study reported that inflammation in the lungs was inhibited by the induction of RSV-specific CD8^+^ IFN-γ^+^ cells [15]. In present study, local immunological responses were not examined. Although the induction of secretory IgA and its responses play important roles against infections, the aim of the present study was to investigate the memory of the immune system following repetitive infections. Regarding local cellular immunity, CD8^+^ IFN-γ^+^ cells were detected in the lungs of cotton rats immunized with a recombinant measles virus expressing the RSV-F, N, and M2-1 proteins after an RSV challenge. Lung tissues were stained using an antibody against cotton rat CD8, and the peribronchial region was examined. A large number of CD8^+^ cells were observed, and may play roles in clearance (data not shown).

## Conclusions

No immune response was elicited after the first infection, and RSV-specific antibodies and CD8^+^ cells were observed after the second infection. Cross-protective NT antibodies were also detected one month after the third infection and thereafter. Furthermore, viral clearance was enhanced by repetitive infections. These results suggest that the induction of humoral and cellular immune responses is effective against RSV infections.

## References

[1] Falsey AR, Hennessey PA, Formica MA, Cox C, Walsh EE. Respiratory syncytial virus infection in elderly and high-risk adults. N Engl J Med. 2005;352: 1749–1759. 10.1056/NEJMoa043951 15858184

[2] Cherukuri A, Patton K, Gasser RA, Zuo F, Woo J, Esser MT, et al Adults 65 years old and older have reduced numbers of functional memory T cells to respiratory syncytial virus fusion protein. Clin Vaccine Immunol. 2013;20: 239–247. 10.1128/CVI.00580-12 23239796 PMC3571266

[3] Nair H, Nokes DJ, Gessner BD, Dherani M, Madhi SA, Singleton RJ, et al Global burden of acute lower respiratory infections due to respiratory syncytial virus in young children: a systematic review and meta-analysis. Lancet. 2010;375: 1545–1555. 10.1016/S0140-6736(10)60206-1 20399493 PMC2864404

[4] Zomer-Kooijker K, Van Der Ent CK, Ermers MJJ, Uiterwaal CSPM, Rovers MM, Bont LJ. Increased risk of wheeze and decreased lung function after respiratory syncytial virus infection. PLoS One. 2014;9: e87162 10.1371/journal.pone.0087162 24498037 PMC3909049

[5] Legg JP, Hussain IR, Warner JA, Johnston SL, Warner JO. Type 1 and type 2 cytokine imbalance in acute respiratory syncytial virus bronchiolitis. Am J Respir Crit Care Med. 2003;168: 633–639. 10.1164/rccm.200210-1148OC 12773328

[6] Chang J, Braciale TJ. Respiratory syncytial virus infection suppresses lung CD8+ T-cell effector activity and peripheral CD8+ T-cell memory in the respiratory tract. Nat Med. 2002;8: 54–60. 10.1038/nm0102-54 11786907

[7] Evans JE, Cane PA, Pringle CR. Expression and characterisation of the NS1 and NS2 proteins of respiratory syncytial virus. Virus Res. 1996;43: 155–161. 10.1016/0168-1702(96)01327-5 8864205

[8] González PA, Prado CE, Leiva ED, Carreño LJ, Bueno SM, Riedel C a, et al Respiratory syncytial virus impairs T cell activation by preventing synapse assembly with dendritic cells. Proc Natl Acad Sci U S A. 2008;105: 14999–15004. 10.1073/pnas.0802555105 18818306 PMC2567482

[9] Prince GA, Horswood RL, Chanock RM. Quantitative aspects of passive immunity to respiratory syncytial virus infection in infant cotton rats. J Virol. 1985;55: 517–520. 4020957 10.1128/jvi.55.3.517-520.1985 PMC254995

[10] Murphy BR, Graham BS, Prince GA, Walsh EE, Chanock RM, Karzon DT, et al Serum and nasal-wash immunoglobulin G and A antibody response of infants and children to respiratory syncytial virus F and G glycoproteins following primary infection. J Clin Microbiol. 1986;23: 1009–1014. 3754878 10.1128/jcm.23.6.1009-1014.1986 PMC268782

[11] Senft AP, Taylor RH, Lei W, Campbell SA, Tipper JL, Martinez MJ, et al Respiratory syncytial virus impairs macrophage IFN-α/β- and IFN-γ-stimulated transcription by distinct mechanisms. Am J Respir Cell Mol Biol. 2010;42: 404–414. 10.1165/rcmb.2008-0229OC 19502390 PMC2848734

[12] Curtis SJ, Ottolini MG, Porter DD, Prince GA. Age-dependent replication of respiratory syncytial virus in the cotton rat. Exp Biol Med (Maywood). 2002;227: 799–802. 12324660 10.1177/153537020222700912

[13] Bueno SM, González PA, Cautivo KM, Mora JE, Leiva ED, Tobar HE, et al Protective T cell immunity against respiratory syncytial virus is efficiently induced by recombinant BCG. Proc Natl Acad Sci U S A. 2008;105: 20822–20827. 10.1073/pnas.0806244105 19075247 PMC2634951

[14] Olson MR, Hartwig SM, Varga SM. The number of respiratory syncytial virus (RSV)-specific memory CD8 T cells in the lung is critical for their ability to inhibit RSV vaccine-enhanced pulmonary eosinophilia. J Immunol. 2008;181: 7958–7968. 19017987 10.4049/jimmunol.181.11.7958 PMC2587004

[15] Yamaji Y, Nakayama T. Recombinant measles viruses expressing respiratory syncytial virus proteins induced virus-specific CTL responses in cotton rats. Vaccine. Elsevier Ltd; 2014;32: 4529–4536. 10.1016/j.vaccine.2014.06.024 24951869

[16] McKimm-Breschkin JL. A simplified plaque assay for respiratory syncytial virus—Direct visualization of plaques without immunostaining. J Virol Methods. 2004;120: 113–117. 10.1016/j.jviromet.2004.02.020 15234816

[17] Sawada A, Komase K, Nakayama T. AIK-C measles vaccine expressing fusion protein of respiratory syncytial virus induces protective antibodies in cotton rats. Vaccine. Elsevier Ltd; 2011;29: 1481–1490. 10.1016/j.vaccine.2010.12.028 PMC7127509 21185852

[18] Martinez-Sobrido L, Gitiban N, Fernandez-Sesma A, Cros J, Mertz SE, Jewell NA, et al Protection against respiratory syncytial virus by a recombinant Newcastle disease virus vector. J Virol. 2006;80: 1130–1139. 10.1128/JVI.80.3.1130-1139.2006 16414990 PMC1346968

[19] Heidema J, de Bree GJ, De Graaff PMA, van Maren WWC, Hoogerhout P, Out TA, et al Human CD8(+) T cell responses against five newly identified respiratory syncytial virus-derived epitopes. J Gen Virol. 2004;85: 2365–2374. 10.1099/vir.0.80131-0 15269378

[20] Rammensee H, Bachmann J, Emmerich NP, Bachor O a, Stevanović S. SYFPEITHI: database for MHC ligands and peptide motifs. Immunogenetics. 1999;50: 213–219. 10.1007/s002510050595 10602881

[21] Shaw CA, Galarneau JR, Bowenkamp KE, Swanson KA, Palmer GA, Palladino G, et al The role of non-viral antigens in the cotton rat model of respiratory syncytial virus vaccine-enhanced disease. Vaccine. Elsevier Ltd; 2013;31: 306–312. 10.1016/j.vaccine.2012.11.006 23153444

[22] Smith G, Raghunandan R, Wu Y, Liu Y, Massare M, Nathan M, et al Respiratory Syncytial Virus Fusion Glycoprotein Expressed in Insect Cells Form Protein Nanoparticles That Induce Protective Immunity in Cotton Rats. PLoS One. 2012;7 10.1371/journal.pone.0050852 PMC3511306 23226404

[23] Glenn GM, Fries LF, Smith G, Kpamegan E, Lu H, Guebre-Xabier M, et al Modeling maternal fetal RSV F vaccine induced antibody transfer in guinea pigs. Vaccine. Elsevier Ltd; 2015; 10.1016/j.vaccine.2015.08.039 26319066

[24] Zeng R, Zhang Z, Mei X, Gong W, Wei L. Protective effect of a RSV subunit vaccine candidate G1F/M2 was enhanced by a HSP70-Like protein in mice. Biochem Biophys Res Commun. Elsevier Inc.; 2008;377: 495–499. 10.1016/j.bbrc.2008.10.002 18851947

[25] Elango N, Prince GA, Murphy BR, Venkatesan S, Chanock RM, Moss B. Resistance to human respiratory syncytial virus (RSV) infection induced by immunization of cotton rats with a recombinant vaccinia virus expressing the RSV G glycoprotein. Proc Natl Acad Sci U S A. 1986;83: 1906–1910. 10.1073/pnas.83.6.1906 3513191 PMC323193

